# Smoking behaviour and passive smoke exposure of adults – Results from GEDA 2019/2020-EHIS

**DOI:** 10.25646/10291

**Published:** 2022-09-14

**Authors:** Anne Starker, Ronny Kuhnert, Jens Hoebel, Almut Richter

**Affiliations:** Robert Koch Institute, Berlin Department of Epidemiology and Health Monitoring

**Keywords:** SMOKING, PASSIVE SMOKING, ADULTS, EDUCATION, GEDA 2019/2020-EHIS

## Abstract

**Background:**

Smoking is a significant health risk and the leading cause of premature death. Passive smoke causes the same negative effects on health as smoking, albeit to a lesser extent. The reduction of tobacco consumption and the protection against passive smoke are thus important health objectives.

**Methods:**

The study German Health Update (GEDA 2019/2020-EHIS) is a cross-sectional telephone survey (04/2019 to 09/2020) of the resident population in Germany with questions relating to the current smoking behaviour and relating to the passive smoke exposure. The analysis sample comprises 22,708 persons from 18 years of age.

**Results:**

24.0% of women and 33.9% of men from 18 years of age smoke currently, at least occasionally. Among both sexes, adults from 65 years of age smoke significantly more rarely than adults in the younger age groups. 4.1% of adults, who do not smoke themselves, are subjected daily to passive smoke exposure indoors. This affects in particular young adults and men. There are educational differences in tobacco consumption and in passive smoke exposure to the disadvantage of adults from lower educational groups.

**Conclusions:**

In Germany, there is still a need for action for effective measures for tobacco prevention, smoking cessation and tobacco control policy, which are effective in all population groups and which take into account the concerns of socially disadvantaged groups.

## 1. Introduction

In the industrialised nations, smoking tobacco is the most significant avoidable health risk and the leading cause of premature death [1]. Cardiovascular, respiratory and cancer diseases occur to an greater extent among smokers. For instance, approximately 15% of all cancers in Germany are attributed to smoking [2]. Lung cancer is thereby the most common tobacco-associated cancer. However, malignant growths on lips, oral cavity, pharynx, oesophagus, larynx and the efferent urinary tract are considered to be tobacco-related diseases. For these cancer diagnoses, at least one-third of all cases in Germany can be attributed to tobacco consumption. Furthermore, smoking has a negative effect on the immune system, metabolism, skeleton, periodontium, eyes and fertility [3]. According to estimates, approximately 127,000 people died as a result of smoking in Germany in 2018 [4]. The costs for treating illnesses and health problems of smoking-related diseases were estimated to be approximately 30 billion euros [5].

Smoking tobacco does not only have negative health consequences for the smokers themselves, but also for persons, who are subjected to a passive smoke exposure. The composition of the tobacco smoke in the ambient air hardly differs from the cigarette smoke, which is inhaled when actively smoking [6]. This is why passive smoking has the same negative health consequences as tobacco smoking, albeit to a lesser extent [7]. Due to the fact that tobacco smoke deposits on surfaces, and harmful substances are released into the ambient air again from there, thirdhand smoke is also responsible for a passive smoke exposure [8]. Due to their increased breathing rate [9] and the not yet fully-developed detoxification system [10], children are particularly at risk when they are confronted with tobacco smoke. Passive smoking during pregnancy endangers the healthy development of the embryo, and passive smoke increases the risk for the sudden infant death among babies [11]. The cost of illness expenses for the passive smoke exposure for persons who live in the same household as smokers, are currently estimated to be 1.3 billion euros per year [12]. The most recently available estimation for the passive smoke-related mortality in Germany from 2003 assumes 3,300 yearly cases of death [7]. It is assumed that the federal and state non-smoking acts enacted since 2007 have contributed to the reduction of the passive smoke exposure and the consequences thereof [1].

In light of the foregoing, the long-lasting and target group-specific reduction of the tobacco consumption including the reduction of the passive smoke exposure represents an important health objective. Since the early 2000s, Germany has been launching various measures to reduce the tobacco consumption among the public, for example multi-level tobacco duty increases or federal and state non-smoking acts. In contrast to other countries, however, Germany does not have a strategy for a long-lasting tobacco control [13], such as, for example, Finland [14], England [15] or Ireland [16]. This is why in 2021, the German Cancer Research Centre (DKFZ), together with more than 50 further health organisations, presented a ‘strategy for a tobacco-free Germany 2040’ [17] with the objective that fewer than five percent of adults and fewer than two percent of adolescents consume tobacco products or electronic cigarettes by then. Currently, however, a comparatively large percentage of adults still smoke and there is still a need for effective measures to curb the tobacco consumption.

To be able to assess current developments and trends in the smoking behaviour and the passive smoke exposure among the public, a frequent monitoring using studies that are representative nationwide is required. The present article thus describes current cross-sectional results on smoking behaviour and passive smoke exposure of adults from GEDA 2019/2020-EHIS, analyses differences with regard to sex, age and education in terms of health inequalities and classifies the results and developments.

**GEDA 2019/2020-EHIS**

Fifth follow-up survey of the German Health Update

**Data holder:** Robert Koch Institute

**Objectives:** Provision of reliable information on the health status, health behaviour and health care of the population living in Germany, with the possibility of European comparisons

**Study design**: Cross-sectional telephone survey

**Population:** German-speaking population aged 15 and older living in private households that can be reached via landline or mobile phone

**Sampling:** Random sample of landline and mobile telephone numbers (dual-frame method) from the ADM sampling system (Arbeitskreis Deutscher Markt- und Sozialforschungsinstitute e.V.)

**Sample size:** 23,001 respondents

**Study period:** April 2019 to September 2020

**GEDA survey waves:**

▶ GEDA 2009
▶ GEDA 2010
▶ GEDA 2012
▶ GEDA 2014/2015-EHIS
▶ GEDA 2019/2020-EHIS

Further information in German is available at www.geda-studie.de

## 2. Methods

### 2.1 Study design and sample

German Health Update (GEDA) is a nationwide telephone cross-sectional survey of the resident population in Germany based on a random sample of landline and mobile phone numbers (dual-frame process). The telephone sampling system of the Arbeitskreis Deutscher Markt- und Sozialforschungsinstitute e.V. (ADM) was used for sampling procedure [18]. The fifth follow-up survey, GEDA 2019/2020-EHIS, took place between April of 2019 and September of 2020. A programmed, fully-structured questionnaire (computer assisted telephone interview, CATI) was used, the questionnaire contents were specified by the European Health Interview Survey (EHIS) [19]. As a whole, complete interviews of 23,001 persons from 15 years of age are available in GEDA 2019/2020-EHIS. An in-depth description of the methodology of GEDA 2019/2020-EHIS can be found in the article German Health Update (GEDA 2019/2020-EHIS) – Background and methodology in the issue 3/2021 of the Journal of Health Monitoring [20].

The information relating to the sex at birth and to the gender identity was used in GEDA 2019/2020-EHIS to describe gender differences: The respondents could specify which sex was registered on their birth certificate and wich gender they feel they belong to. For the analyses by gender, those who were born as women or men, respectively and also identify as such, as well as transgender people, who assign themselves to one of these two genders. Persons who do not identify as female or male (gender-diverse people) are not shown due to the small number of cases, but they are included in the total category [21].

The analyses relating to the smoking behaviour are based on valid information from a total of 22,699 participating persons from 18 years of age, those relating to the passive smoke exposure relate to the valid information of a total of 17,823 persons from 18 years of age, who do not smoke themselves.

### 2.2 Instruments and indicators

#### Smoking behaviour

To record the smoking status, the participants of GEDA 2019/2020-EHIS were asked the question: ‘Do you smoke any tobacco products, including heated tobacco products? Please exclude electronic cigarettes or similar electronic devices.’ (Response category: ‘Yes, daily’, ‘Yes, occasionally’, ‘No longer’ and ‘I have never smoked’). Based on the response categories, a differentiation will be made below between current smokers (daily or occasionally), former smokers as well as never-smokers.

#### Passive smoke exposure

The passive smoke exposure was surveyed with the following question: ‘How often are you exposed to tobacco smoke indoors?’ (Response category: ‘Every day, 1 hour or more a day’, ‘Every day, less than 1 hour per day’, ‘At least once a week (but not every day)’, ‘Less than once a week’ and ‘Never or almost never’). The daily passive smoke exposure can be assessed based on the response categories (less than 1 hour or 1 hour or more). This information relates to persons who do not smoke themselves.

### 2.3 Statistical analyses

To correct deviations of the sample from the population structure, the analyses are performed with a weighting factor. As part of the data weighting, a design weighting is made initially for the different selection probabilities (mobile phone and landline) and an adaptation is made subsequently to the official population figures based on age, sex, state and district type (as of: 31.12.2019). In addition, adjustment is made for the education distribution identified by the 2017 Microcensus [22]. This was conducted in accordance to the International Standard Classification of Education (ISCED) [23].

The descriptive analyses of the tobacco consumption differentiate by the variables gender, age (18 to 29 years of age, 30 to 44 years of age, 45 to 64 years of age, from 65 years of age) and education (ISCED: low education group (ISCED stage 1 and 2), medium education group (ISCED stage 3 and 4), high education group (ISCED stage 6 and 8)) [23] and is made by the calculation of prevalence (relative frequencies) with 95% confidence intervals (95% CI). A significant difference between groups (determined by means of Chi-square test) is assumed when the p value, which is determined in consideration of the weighting and the survey design, is less than 0.05.

Due to small cell sizes and the associated larger statistical uncertainty, the descriptive analyses of the sample for the passive smoke exposure differentiate by gender and age as well as by gender and education.

The analyses were performed using Stata 17.0. To appropriately factor in the weighting when calculating confidence intervals and p values, all analyses were calculated using survey procedures.

## 3. Results

### 3.1 Smoking behaviour

According to the self-reported information from GEDA 2019/2020-EHIS, 28.9% of adults in Germany smoke at least occasionally, this figure is 24.0% among women and 33.9% among men. More than half of the women (52.4%) states never having smoked. This figure is 36.1% among the men (Table 1). The percentage of the current smokers differs relatively little in the age groups of up to 64 years of age. A significant decline can only be observed starting at the age of 65. If the percentage of smokers in the individual age groups is examined by education, it is larger in the low and in the medium education group than in the high education group, among women as well as among men. People 65 years of age and older, where no education differences exist, form the exception among both sexes.

### 3.2 Passive smoke exposure

Currently, 4.1% of the non-smoking adult population in Germany is subjected daily to passive smoke exposure (Table 2), together with those who are affected by it at least once per week, this figure is 8.2%. Women are less likely than men to be affected by daily or even weekly passive smoke exposure. The highest exposure is reflected among young adults between the ages of 18 and 29. The passive smoke exposure decreases with increasing age, in particular in the age group of 65 and older.

Among men, the data from the study GEDA 2019/2020-EHIS shows differences in the daily passive smoke exposure, depending on the education level of the respondents: Men from the low and from the medium education group are more frequently affected by a daily passive smoke exposure indoors than those from the high education group.

## 4. Discussion

According to the data from GEDA 2019/2020-EHIS, 28.9% of adults, proportionately more men than women, currently still smoke (daily or occasionally) in Germany. It is only in the group aged 65 and older that the proportion of smokers and the educational differences decrease.

8.2% of the non-smoking adults are frequently subjected to passive smoke exposure. A significant sex difference can also be observed here to the effect that men are affected more frequently. A frequent passive smoke exposure is reported in particular by women and men between the ages of 18 and 29, and even further by men up to the age of 44. Among men, there are also strong differences in education to the disadvantage of the low education group.

With regard to their size, the present results relating to the smoking behaviour fall within the results of other nationwide representative surveys and studies. It is important to take into consideration that the surveys are partly older, partly refer to different age ranges and that different survey methodologies (survey by telephone, paper questionnaire or online) or survey tools were used, respectively, which limits a direct comparison. According to the data from the socioeconomic panel (SOEP) with the question ‘Do you currently smoke, either cigarettes, pipes or cigars?’ (Response options: No, Yes), the percentage of smokers in 2016 among persons from 18 years of age is 22.4% for women, 29.5% for men [24]. According to the sample census 2017 (question: ‘Do you currently smoke?’ (Response options: Yes, regularly, yes, occasionally, no), 19% of the women from 15 years of age and older and 26% of the men of the same age smoke [25]. According to data from the epidemiological surveys on addiction 2018, which relate to the population between the ages of 18 and 24, 20.0% of women and 26.4% of men indicate having smoked a cigarette, cigar, cigarillo or pipe within the last 30 days prior to the survey [26], and in the current survey of the German Study on Tobacco Use (DEBRA) in 2020/2021, which includes persons from the age of 14, 26.0% of women and 34.0% of men smoke [27]. The question was: ‘Which of the following statuses best describes you? Please note that this refers to smoking tobacco and not electronic cigarettes or tobacco heaters.’ Response options: I smoke cigarettes, namely daily., I smoke cigarettes, but not daily., I do not smoke cigarettes at all, but I smoke tobacco in another form (for example pipe or cigar)., I have stopped smoking completely in the last 6 months., I have stopped smoking completely more than 6 months ago., I never smoked (never longer than one year).

The passive smoke exposure in the last seven days of persons from the age of 14 in vehicles, indoors and outdoors was surveyed in two waves of the DEBRA study (between January and March of 2020) [28]. According to this, 25% of respondents indicate an exposure indoors in the last seven days. 11.4% indicated an exposure on one to two days in the last seven days. Among non-smokers, this percentage was 9.2%, among former smokers it was 11.1%. Due to the different question, the results can only be partially compared with those of GEDA 2019/2020-EHIS. However, they lie within a similar range.

The result of GEDA 2019/2020-EHIS, according to which more men than women smoke, can also be observed in the already mentioned studies. This sex difference has partly historical reasons because smoking in Germany among women was less socially acceptable than among men until the 1960s, and only a small percentage of women started smoking [29]. This is also confirmed by analyses relating to trends and developments in the tobacco consumption of adults in Germany, which show a continuous increase of the prevalence of ever having smoked among women born between 1930 and 1959, while the percentage among men has hardly changed in this time period [30]. The analysis was therefore also able to show that the sex difference reduced over time [30]. This is attributed to a sharper decline of the smoking prevalence among men compared to women from 2003 onward. The percentage of people who have never smoked since the 1970s can be illustrated using the data from the Drug Affinity Study conducted by the Federal Centre for Health Education, and can thus show, how accepted and widespread smoking is among adolescents and young adults and whether sex differences exist or the development thereof, respectively. The percentage of never-smokers increases continuously among adolescents between the ages of 12 and 17, and, with 85.1% in 2019, is as high as never before, whereby there are hardly any sex differences any longer [31]. Among young adults between the ages of 18 and 25, an increase of the percentage of never-smokers can also be observed, it is currently 45.9%. However, there are differences by sex (women: 50.5%, men: 41.9%) in these age groups. They can in particular be attributed to differences among the 22- to 25-year olds [31]. These results can be considered to be an indication that the sex differences will equalise further in the future when birth cohorts that started smoking at a lower percentage, become middle-aged and of more advanced age.

There is also a gender difference in passive smoke exposure in that men are more often affected. The earlier GEDA studies already showed differences in the passive smoke exposure to the disadvantage of men [32, 33]. The DEBRA study finds sex differences for the passive smoke exposure in vehicles, but not indoors or outdoors [28]. According to this, the probability is lower among women that they were exposed to passive smoking in a vehicle in the last seven days.

The fact that the percentage of smokers decreases only with increasing age (from 65 years of age) is also substantiated in the other studies relating to the smoking behaviour in Germany [24, 25]. The reason for the decline could be cohort effects because the smoking behaviour of previous birth cohorts differs from that of subsequent birth cohorts [29, 30]. A further reason could be the diseases and symptoms that occur more frequently in the higher age groups, which is why smoking is given up. The increased premature mortality of smokers can also contribute to the percentage of smokers being lower among older population groups [34–36]. The age differences in the passive smoke exposure, according to which in particular young adults are affected, are also shown in other studies [28, 32, 37].

A higher tobacco consumption in low education groups in Germany has been noticeable for many years [38–40]. The present results from GEDA 2019/2020-EHIS confirm this. The development over time makes it clear that the differences in the smoking behaviour have increased starting in the early 2000s to the effect that there has been a significant decline in the percentage of smokers among women and men of the high education group, whereas the percentage remained approximately the same among men in the low education group, and even increased among women [38, 40]. Educational differences in the smoking behaviour already become apparent during adolescence [30, 41], a formative period in the decision for or against smoking. This behaviour manifests itself in young adulthood. According to this, the higher smoking prevalence in the low education groups is characterised by a more frequent smoking initiation and rarer cessation [42].

In the case of the passive smoke exposure, educational differences also become apparent to the disadvantage of the low education group, in particular among men between the ages of 30 and 64. In GEDA 2014/2015-EHIS, where in addition to the extent, the location of the passive smoke exposure was also surveyed, analyses showed that the higher passive smoke exposure in the low and medium education group among men who do not smoke could mainly be traced back to a high exposure to passive smoke in the workplace [32]. This is consistent with results, according to the which the percentage of smokers is still highest in professions with a low professional status [43], even though the Workplace Ordinance has been regulating non-smokers protection for employees since 2002. According to this, employers are legally obligated to protect non-smoking employees from the health risks caused by passive smoking. The exact structure, however, is not regulated in the ordinance, but is up to the employers. It can currently not be assessed whether men from low education groups are still affected more frequently by passive smoke in the workplace. The DEBRA study also finds educational differences with regard to the passive smoke exposure in vehicles and indoors to the effect that people with low education had a higher risk of being exposed to passive smoke than persons with high education [28].

The smoking behaviour of the adult population is captured routinely in health surveys conducted by the Robert Koch Institute, most recently in GEDA 2014/2015-EHIS [44]. When comparing the results, it appears that there is an increase of the percentage of smokers in the adult population in GEDA 2019/2020 (23.8% v. 28.9%). However, the results of these two survey points can only be compared to a limited extent because different survey modes were used (online questionnaire v. telephone interview) and the questions for capturing the smoking behaviour were changed in the EHIS (while the response categories remained the same). GEDA 2019/2020-EHIS explicitly captures the smoking of tobacco products including tobacco heaters, while GEDA 2014/2015-EHIS asked ‘Do you smoke’. The samples of both surveys also differ: GEDA 2014/2015-EHIS is based on a registration office sample, and GEDA 2019/2020-EHIS is based on a telephone sample.

Earlier trend evaluations from other surveys can thus be used to assess the development in the smoking behaviour of adults. For the time period between 2003 and 2015, they substantiate a decreasing percentage of smokers [30]. Other surveys, such as the sample census 2017 [25] and the epidemiological survey on addiction 2018 [45] had most recently reported decreasing percentages of smokers among the adult population. The DEBRA study recorded a slight increase of the percentage of smokers for the first time again between 2020 and 2021 [27]. A changed smoking behaviour as a result of the COVID-19 pandemic is discussed as one reason [46, 47]. The results from the COSMO study (COVID-19 snapshot monitoring), which also analyses, among other things, the behaviour of the population during the Corona pandemic, suggest that the spread and the frequency of the consumption of tobacco and electronic cigarettes has increased during the pandemic (between April of 2020 and September of 2021) [48]. The design of the current GEDA study allows the assessment of the monthly progression of the smoking behaviour during the entire study period (04/2019 to 09/2020). The analyses do not reveal any noticeable developments in the smoking behaviour during the pandemic [49, 50]. In two waves of the DEBRA study (June to August of 2020), participants were asked specifically about possible behavioural changes in the smoking behaviour as a result of measures to contain the first COVID-19 infection wave in the spring of 2020. The majority of the respondents thereby did not specify any changes in the smoking behaviour. In addition, others reported having smoked more than before or also having reduced the consumption, with the percentage of persons with increasing consumption being larger than the percentage with reduced consumption [47].

The passive smoke exposure was also captured in earlier health surveys conducted by the Robert Koch Institute, most recently in GEDA 2014/2015-EHIS [32]. At that time, 11.3% of the non-smokers reported passive smoke exposure indoors for less than one hour or one hour per day or more [32]. These results, however, can also be compared only to a limited extent because the specifications for the response categories in both EHIS waves were changed, while the questions remained the same. Five response categories (see above) were available in GEDA 2019/2020-EHIS, three were available in GEDA 2014/2015-EHIS (‘Never or almost never, ‘Less than 1 hour per day’, ‘1 hour or more a day’). Even if the change of the response categories limits the comparability, the low prevalence of the daily passive smoke exposure in GEDA 2019/2020-EHIS (4.1%) suggests that this affects fewer people than just five years ago. The fact that the question about the passive smoke exposure does not specify a frame of reference, for example in the last month, is restrictive. It is thus rather an approximate estimation than an exact representation of the exposure.

To reduce the tobacco consumption among the public and to better protect non-smokers against the health risks of passive smoking, tobacco control policy measures have been introduced in Germany since the early 2000s to an increased extent. This includes multi-level tobacco duty increases, the revision of the Workplace Ordinance with the regulation of the protection of the non-smoking employees while working, the increase of the age limit for purchasing and consuming tobacco products from 16 to 18, the federal and state non-smoker protection acts, and the tightening of the legal regulations for tobacco advertisement [51]. In addition, target group- and setting-related programs and campaigns were started. Significant impulses for this development originated from the Framework Convention on Tobacco Control (FCTC), the stated aim of which is the protection against health-related, social, ecological and economic consequences of tobacco consumption and of passive smoking. The Convention is a treaty under international law that was adopted by the 56th World Health Assembly and entered into force on February 27, 2005. Germany signed the treaty in 2004, thereby committing itself to ratification.

By international standards, the measures to control tobacco implemented in Germany by 2019 were still relatively weak. This assessment was also reflected in the European tobacco control scale of 2019, for which a total of 36 countries were compared with regard to their efforts to effectively prevent and control tobacco. Germany was in last place in this ranking [52]. Since 2019, there have then been further legal measures, such as those to limit tobacco advertisement in movie theatres and to gradually introduce the ban on outdoor advertising based on the changes to the Tobacco Products Act of 2020, and a change to the Tobacco Duty Law and the Tobacco Duty Ordinance based on the Tobacco Duty Modernisation Act of 2021, which regulates a taxation of cigarettes and fine cut in the next five years, as well as the adaptation of the taxation of heated tobacco and substances consumed in electronic cigarettes, including water pipe tobacco. The ‘Strategy for a tobacco-free Germany 2040’ was likewise published in 2021 by a broad alliance of health and civic organisations [17]. The goal is that in 2040, less than five percent of adults and less than two percent of adolescents in Germany consume tobacco products, electronic cigarettes or other related products, in particular if they contain the addictive nicotine. The strategy names ten measures with concrete partial steps and time targets for the implementation thereof, which are based on the FCTC. In detail, they are the following measures: Tobacco duty increase, support for smokers when quitting smoking, advertising ban and standardised packaging, reduction of the availability of tobacco and related products, protection against passive smoke exposure, implementation of the children’s rights and protection of minors with regard to tobacco, educational campaigns, initiatives for the control of tobacco and alternatives for tobacco growing as part of the development cooperation, protecting political decisions and the organisations thereof against being influenced by the tobacco industry as well as frequent review, adaptation and further development of the mentioned measures. From a public health perspective, the health policy-related implementation of these measures would be an important contribution in order to further decrease the most significant avoidable health risk in Germany and to take thereby also into greater account the concerns of socially disadvantaged groups.

## Key statement

- 24.0% of women and 33.9% of men aged 18 years and older smoke at least occasionally.
- Adults aged 65 years and over smoke significantly less often than adults in the younger age groups.
- 8.3% of non-smoking adults are regularly exposed to passive smoke exposure, 4.1% daily.
- Young adults are most frequently affected by passive smoke exposure.
- Smoking and passive smoke exposure are significantly more widespread in the lower than in the high educational group.

## Figures and Tables

**Table 1:** Smoking status by gender, age and education (n=11,955 women, n=10,682 men) Source: GEDA 2019/2020-EHIS

	Smoking					
	Daily or occasionally		No longer		Never	
	%	(95% CI)	%	(95% CI)	%	(95% CI)
Total	28.9	(27.9–29.9)	26.7	(25.9–27.5)	44.4	(43.4–45.4)
**Women**	**24.0**	**(22.7–25.3)**	**23.6**	**(22.5–24.7)**	**52.4**	**(51.1–53.8)**
**18–29 years**	30.4	(26.4–34.6)	12.2	(9.7–15.4)	57.4	(53.1–61.6)
Low education group	47.4	(36.7–58.4)	10.7^*^	(5.3–20.2)	41.9	(31.8–52.7)
Medium education group	29.1	(24.2–34.6)	13.6	(10.2–17.9)	57.3	(51.7–62.8)
High education group	18.7	(13.3–25.5)	9.6	(6.5–14.0)	71.8	(64.7–77.9)
**30–44 years**	29.6	(26.6–32.8)	23.5	(21.0–26.1)	46.9	(43.8–50.0)
Low education group	46.1	(33.7–59.0)	15.5^*^	(8.1–27.6)	38.4	(26.8–51.4)
Medium education group	32.7	(28.7–37.0)	24.9	(21.4–28.7)	42.4	(38.2–46.7)
High education group	17.1	(14.2–20.3)	24.4	(21.5–27.6)	58.5	(54.8–62.2)
**45–64 years**	28.2	(26.3–30.3)	27.2	(25.5–29.1)	44.5	(42.5–46.5)
Low education group	36.6	(29.5–44.3)	28.0	(21.7–35.3)	35.4	(28.4–43.1)
Medium education group	29.4	(27.1–31.9)	27.2	(25.0–29.5)	43.4	(41.0–45.9)
High education group	18.8	(16.9–20.9)	27.0	(24.9–29.2)	54.2	(51.7–56.7)
**≥65 years**	11.3	(9.8–12.9)	25.2	(23.3–27.2)	63.6	(61.3–65.8)
Low education group	11.7	(8.5–15.9)	20.4	(16.5–24.9)	67.9	(62.6–72.7)
Medium education group	11.2	(9.7–13.0)	27.4	(25.2–29.7)	61.4	(58.9–63.9)
High education group	10.3	(8.7–12.2)	29.0	(26.5–31.7)	60.7	(57.8–63.4)
**Men**	**33.9**	**(32.5–35.4)**	**29.9**	**(28.7–31.2)**	**36.1**	**(34.8–37.5)**
**18–29 years**	40.5	(36.7–44.3)	13.1	(10.6–15.9)	46.5	(42.8–50.2)
Low education group	49.7	(41.4–58.1)	9.2^*^	(5.3–15.5)	41.1	(33.4–49.3)
Medium education group	40.4	(35.5–45.6)	13.1	(9.8–17.2)	46.5	(41.6–51.5)
High education group	26.9	(21.8–32.7)	18.5	(14.0–24.1)	54.5	(48.3–60.6)
**30–44 years**	45.0	(41.7–48.4)	23.0	(20.5–25.8)	32.0	(29.2–34.8)
Low education group	57.6	(45.0–69.2)	23.3^*^	(14.7–34.9)	19.2^*^	(11.2–30.8)
Medium education group	52.3	(47.6–57.0)	21.8	(18.3–25.8)	25.9	(22.2–29.9)
High education group	28.5	(25.2–32.1)	24.7	(21.6–28.0)	46.8	(43.2–50.4)
**45–64 years**	36.7	(34.4–39.1)	31.2	(29.1–33.3)	32.2	(30.1–34.2)
Low education group	47.6	(37.6–57.9)	24.7	(17.2–34.1)	27.7	(19.7–37.4)
Medium education group	42.1	(38.8–45.4)	32.9	(29.9–36.0)	25.0	(22.4–27.9)
High education group	23.3	(21.3–25.5)	30.7	(28.6–32.9)	46.0	(43.6–48.4)
**≥65 years**	13.6	(11.7–15.8)	47.8	(45.2–50.5)	38.6	(36.0–41.2)
Low education group	20.8^*^	(12.1–33.4)	40.0	(29.4–51.7)	39.1	(28.2–51.3)
Medium education group	13.5	(11.0–16.4)	49.8	(46.0–53.6)	36.7	(33.1–40.5)
High education group	11.5	(10.0–13.2)	47.4	(44.9–49.9)	41.1	(38.7–43.6)

CI=confidence interval,

^*^Number of cases is n<20

**Table 2:** Passive smoke exposure of non-smokers by gender, age and education (n=9,695 women, n=8,083 men) Source: GEDA 2019/2020-EHIS

	Passive smoke exposure							
	Daily		At least once a week		Less than once a week		Never or almost never	
	%	(95% CI)	%	(95% CI)	%	(95% CI)	%	(95% CI)
Total	4.1	(3.7–4.7)	4.1	(3.–4.7)	5.8	(5.3–6.3)	85.9	(85.1–86.7)
**Women**	**3.0**	**(2.5–3.7)**	**2.9**	**(2.4–3.6)**	**4.7**	**(4.1–5.3)**	**89.4**	**(88.3–90.3)**
**Age group**								
18–29 years	7.2	(4.9–10.6)	9.0	(6.3–12.8)	11.0	(8.6–14.0)	72.7	(68.0–76.9)
30–44 years	3.2	(2.1–4.8)	2.6	(1.6–4.0)	5.6	(4.2–7.5)	88.7	(86.1–90.8)
45–64 years	2.7	(2.0–3.7)	2.5	(1.7–3.5)	3.7	(2.9–4.6)	91.1	(89.6–92.5)
≥65 years	1.5	(1.0–2.2)	1.2	(0.8–1.7)	2.5	(1.9–3.3)	94.8	(93.7–95.7)
**Education level**								
Low education group	3.9	(2.4–6.1)	1.7^*^	(0.9–3.2)	3.7	(2.4–5.9)	90.7	(87.7–93.0)
Medium education group	3.2	(2.5–4.1)	3.9	(3.0–4.9)	4.6	(3.9–5.5)	88.3	(86.8–89.6)
High education group	1.8	(1.4–2.5)	1.5	(1.1–2.1)	5.7	(4.8–6.9)	90.9	(89.5–92.1)
**Men**	**5.3**	**(4.5–6.3)**	**5.6**	**(4.8–6.5)**	**7.2**	**(6.4–8.0)**	**81.9**	**(80.6–83.2)**
**Age group**								
18–29 years	9.1	(6.5–12.5)	10.1	(7.6–13.4)	13.6	(10.9–16.8)	67.2	(62.7–71.4)
30–44 years	6.5	(4.7–8.9)	8.8	(6.6–11.7)	10.0	(8.0–12.5)	74.7	(71.0–78.0)
45–64 years	5.8	(4.4–7.6)	4.3	(3.2–5.7)	5.8	(4.7–7.0)	84.1	(81.8–86.2)
≥65 years	2.1	(1.6–2.9)	2.5	(1.7–3.7)	3.6	(2.8–4.6)	91.8	(90.2–93.1)
**Education level**								
Low education group	9.8	(6.3–15.0)	8.6	(5.4–13.4)	5.5	(3.4–9.0)	76.0	(69.7–81.4)
Medium education group	6.2	(5.1–7.6)	5.9	(4.8–7.3)	7.5	(6.3–8.8)	80.4	(78.3–82.3)
High education group	2.5	(2.0–3.3)	4.0	(3.3–4.9)	7.4	(6.5–8.4)	86.0	(84.6–87.2)

CI=confidence interval,

^*^Number of cases is n<20
